# Interventions that address food insecurity for children aged 0–11 years, families, and pregnant women in the UK: a systematic review of intervention studies

**DOI:** 10.1017/jns.2026.10111

**Published:** 2026-06-11

**Authors:** Caitlin Holt, Nicola Heslehurst, Ruth Kipping, Michael P. Daly, Zoe Bell, Andrew Mahon, Patsy Temple, Alice Porter

**Affiliations:** 1 Population Health Sciences, Bristol Medical School, University of Bristol, Bristol, UK; 2 Population Health Sciences Institute, Faculty of Medical Sciences, Newcastle University, Newcastle-Upon-Tyne, UK; 3 The Centre for Translational Research in Public Health, Fuse, Newcastle-Upon-Tyne, UK; 4 Department of Nutritional Sciences, King’s College London, London, UK; 5 Somerset Council Public Health, Somerset County Council, Somerset, UK; 6 NIHR Bristol Biomedical Research Centre, University Hospitals Bristol and Weston NHS Foundation Trust and University of Bristol, Bristol, UK

**Keywords:** Children, Families, Food utilisation, Food accessibility, Food availability, Food insecurity, Food security, Food stability, Infants, Intervention, Pregnant

## Abstract

Food insecurity (FI) has increased in recent years due to economic shifts and rising food prices, with 13.6% of UK households experiencing FI, 47% including children in 2024. Following PRISMA guidelines, this systematic review with narrative synthesis explored the impact of UK interventions addressing FI for children, families, and pregnant women. Seven databases and two clinical trial registers were systematically searched for articles published between 2008–November 2023 (rapid search November 2023–February 2026). Peer-reviewed intervention studies were eligible if they were conducted in the UK, had an experimental design, targeted at least one FI pillar (accessibility, utilisation, availability, and stability) and if most participants were children aged 0–11 years, families with at least one child 0–11 years, or pregnant women. 18,225 articles were identified (rapid search identified 5,514); 11 intervention studies were included (rapid search *n* = 2). Types of interventions included cooking interventions (*n* = 4), free school meals (*n* = 3), holiday clubs (*n* = 2), supermarket vouchers (*n* = 1), and food bags (*n* = 1). Food availability was targeted in seven studies, food accessibility and utilisation in five, and food stability in one, one study included FI as an outcome. The interventions demonstrated barriers and facilitators to inform future intervention development. Most studies were considered serious or critical risk of bias. A lack of high-quality interventions addressing FI were identified. The additional studies reflected the evidence from the original studies. Government policies and funding are needed to relieve FI. Research needs to co-develop acceptable interventions and evaluate their effectiveness to reduce FI among families.

## Introduction

Food security is defined as a situation in which ‘all people, at all times, have physical and economic access to sufficient, safe, and nutritious food that meets their dietary needs and food preferences for an active and healthy life’.^(1)^ Food insecurity (FI) occurs when people do not meet these criteria. FI is currently an important and growing public health issue in the UK, in 2022/2023, 7.2 million people were living in food insecure households, a rise of 2.5 million people since the previous year.^(2)^ The Food Foundation^(3)^ estimates approximately 13.6% (7.2 million adults) of UK households experienced FI in June 2024 and 47% of households experiencing FI include children under the age of 16 years,^(2,4)^ with households with children under 4 years of age most vulnerable to FI.^(3)^ This highlights FI disproportionately affects households with children. Research suggests FI negatively impacts children’s health, wellbeing, and academic outcomes.^(5)^

There are four primary dimensions of food security: (1) availability of food, which is achieved when there is an adequate and consistent source of quality food; (2) access to food, which is achieved when all individuals have sufficient resources, both economic and physical, to source the appropriate food to meet their dietary requirements and food preferences; (3) food utilisation, which is achieved when individuals are able to make suitable use of food, based on food safety and nutritional knowledge, to attain nutritional well-being, whilst also having access to water and sanitation needed for appropriate food preparation hygiene; and (4) stability over time, which is achieved when the previous dimensions are consistent over time and not determined by external factors such as weather and food price changes.^(6)^ Thus, FI occurs when one or more of these dimensions are not achieved.

One of the structural drivers of FI in the UK is rising food prices.^(7)^ In March 2023, food and drink inflation hit a peak of 19.1%, the highest rate since 1977.^(2)^ As a result, the number of people turning to sources of external support (e.g. food banks and pantries) increased. Whilst the use of food aid, such as food banks, has been shown to temporarily mitigate the severity of FI, it cannot eliminate the issue, partly due to limited availability of varied nutritionally dense high-quality food, which is both socially acceptable and culturally appropriate.^(8)^

Nutritional deficiencies and chronic illness have been highlighted as risks associated with FI in children.^(9)^ FI can be detrimental to children’s relationship with food and can lead to unhealthy weight control behaviours, loss of control eating, eating in the absence of hunger, and picky eating.^(10)^ These behaviours have been shown to be associated with a heightened risk of being affected by overweight or obesity.^(9,11)^ Parents and caregivers will often attempt to shield their children from the realities of FI, leaving themselves hungry.^(12)^ Despite this, children are often aware of their food security status and may adopt coping strategies, such as eating less, eating more at school, lack of interest in food, and becoming emotionally numb.^(12)^ As a result, exposure to FI has also been found to be associated with adverse mental health outcomes in children, specifically greater risk of stress, anxiety, and depression.^(11–13)^ FI can also have a detrimental impact on development in children under 5 years^(14)^ and has been shown to have a lasting impact on academic and cognitive outcomes.^(13–15)^ Research suggests this may be due to children being unable to perform at school, lacking concentration and energy due to hunger,^(12)^ which can be further exacerbated by stress and poor mental health.^(15)^

Socio-economic status, material deprivation, and unemployment have all been associated with increased risk of FI.^(5)^ This is partially due to socio-economically deprived households facing more barriers to purchasing and preparing healthy foods, relating to access and affordability.^(16)^ For example, healthy foods are significantly more expensive per calorie compared to less healthy foods.^(17)^ To afford the UK Government’s recommended healthy diet, 40% of the poorest households’ disposable income would need to be spent on healthy food.^(17)^ However, it is vital to recognise the complexities of barriers to healthy eating, including factors such as access to fuel, space, and equipment to cook, which push families to frequently re-prioritise their resources.^(7)^

Due to the rapidly increasing prevalence of FI in the UK, disproportionality affecting households with children, and complex impacts on children and families nutritional, physical, and mental health and wellbeing, it is important to understand what can be done to prevent and alleviate FI among families with children in the UK. FI policies and interventions for children in developed countries were assessed in a systematic review of studies published up to July 2018, which found the evidence was limited in scope and quality.^(18,19)^ A UK-based scoping review explored community interventions for adults in the UK, which similarly found the evidence was limited in scope and quality. Additionally, they found that the nutritional quality of the food provided was poor and failed to assess the interventions impact on FI.^(20)^ There are no reviews of interventions delivered in the UK context focusing on children, families or pregnant women where the context for FI is different to other countries, particularly the US due to differing federal support programmes. The overall aim of the systematic review was to explore the impact of UK interventions that addressed FI for children, families with children and pregnant women, to inform the future design and implementation of interventions to alleviate FI in these populations.

## Methods

### Study design

This systematic review with narrative synthesis was conducted and reported following the Preferred Reporting Items for Systematic Reviews and Meta Analysis Guidelines (PRISMA).^(21)^ The protocol for this review was prospectively registered in the Prospective Register of Systematic Reviews (PROSPERO) (registration number CRD42023453155).

### Objectives

The primary objectives of this review were to determine: 1a. the effect of interventions on alleviating FI; 1b. which FI dimensions are targeted by interventions that addressed FI (availability, accessibility, utilisation, or stability); 2a. the context (e.g. rural or urban) of UK interventions that addressed FI ; 2b. the theory of how and why interventions that addressed FI were expected to have the desired outcomes; 3a. the features of successful interventions on alleviating FI; and 3b. the uptake, participant/ provider acceptability and feasibility of the interventions. A secondary objective was identifying any additional outcomes reported beyond FI, including how they were measured.

### Eligibility criteria

Table 1 presents the eligibility criteria for the study, defined using PICOS (Population, Intervention, Comparison, Outcomes, Study design). The eligible population of children under eleven was chosen as in the UK children finish primary school and move up to secondary school at this age.

**Table 1.** PICOS framework Table 1 long description.

Population	The majority of participants had to be children aged 0–11 years old, families with at least one child aged 0–11 years, or pregnant women.
Intervention	Conducted in the UK. Studies were also required to address at least one of the pillars of FI (accessibility, utilisation, availability or stability) to be eligible for inclusion.
Comparison	We applied no eligibility criteria relating to comparison groups.
Outcomes	We applied no eligibility criteria relating to reported outcomes.
Study design	Peer-reviewed studies were eligible for inclusion if: they were intervention studies with an experimental design, process evaluations of intervention studies (including RCTs, before-and-after-studies, quasi-experimental studies, factorial designs, pilot studies, and feasibility studies) or qualitative studies of an intervention with an experimental design.

Studies were excluded if they focused on children with specialist feeding requirements or chronic medical conditions, or interventions that targeted weight loss. Reviews, editorials, opinion pieces, commentaries, protocols, and grey literature were excluded.

### Search strategy

We conducted a comprehensive systematic literature search of the following seven databases in October 2023: Web of Science, MEDLINE, PsycINFO, EMBASE, CINAHL, Cochrane Library, and ProQuest. Searches were restricted to English language articles published from 2008 onwards to align with when the global financial crisis occurred. A search update was conducted in February 2026 to identify additional articles published between November 2023 and February 2026 in the same seven databases. Our search strategy (Supplementary File 1) included the following search terms: ‘food insecurity’, ‘food availability’, ‘food accessibility’, ‘food utilisation’, ‘food stability’, infant, child, pregnan*, preschool, families, ‘randomized controlled trial’, intervention, program*, policy, ‘before and after’ and quasi*. These were informed by search strategies used in previous systematic reviews.^(18,19,22)^ The search terms and the final strategy were developed in consultation with a subject librarian and were combined using Boolean operators ‘AND’ and ‘OR’. The search strategy did not include any country restrictions. Studies outside the UK were filtered out during the screening stage to ensure all potentially eligible studies were captured.

Clinical trial registers (ClinicalTrials.gov and WHO ICTRP) were searched on the 19 December 2023, to identify additional potentially relevant articles, utilising the same keywords for the database searches (Supplementary File 2). Forward and backward citation searches were conducted for all included studies and relevant reviews using CitationChaser.^(23)^

### Study selection

The primary investigator conducted the searches, saved all searches in an EndNote library, removed duplicates, and exported them to Rayyan for screening.^(24)^

The primary investigator screened the titles and abstracts of the identified studies. This was independently conducted in duplicate. Discrepancies between reviewers were reviewed by the wider research team and resolved by consensus after consulting the protocol. Upon completing the title and abstract screening, the entire review team piloted full-text screening using the inclusion and exclusion criteria on a sample of three reports to refine and clarify the eligibility criteria. Potentially eligible studies were taken forward for full-text screening, and screening was conducted by the primary investigator and independently completed in duplicate. Where clarification was needed to determine if a study was eligible for inclusion (e.g. ages of the children), the primary investigator contacted the study’s authors for additional details. For the search update the primary investigator conducted all the screening stages independently.

### Data extraction

A data extraction form was developed including six overarching categories: study background, population characteristics, intervention characteristics, outcome results, process evaluation results, and economic outcomes. Data extraction was performed by four of the study investigators independently. Any disagreements were resolved by the wider research team. Where missing data were identified, the primary investigator contacted the studies’ authors for additional details.

As part of the data extraction category; intervention characteristics, study investigators were required to assess which food security pillars were addressed in each of the interventions. Table 2 presents the pillars, their definitions used in the decision-making process, and the types of characteristics that were used to assess which of the pillars were addressed in each intervention.

**Table 2.** Intervention characteristics for food security pillars Table 2 long description.

Food security pillars	Definition	Intervention characteristics
Availability	Achieved when there is an adequate and consistent source of quality food.^(6)^	Consistent meals provided Consistent food provision, e.g. ingredients
Accessibility	Achieved when all individuals have sufficient resources, both economic and physical, to source the appropriate food to meet dietary requirements and food preferences.^(6)^	Eliminated factors that prevent participants from engaging with the intervention in the study design. Provided food to the participants’ homes Use of existing known facilities and staff members to the participants for familiarity to increase confidence. Tackled barriers to the time taken to cook in the intervention design. Budgeting skills provided Means to purchase food provided
Utilisation	Achieved when individuals can make suitable use of food, based on food safety and nutritional knowledge to attain nutritional well-being, whilst also having access to water and sanitation needed for appropriate food preparation and hygiene.^(6)^	Cooking skills provided Nutrition knowledge provided Food preparation skills provided Recipes provided
Stability	Achieved when all the previous dimensions can be consistent over time and not determined by external factors such as weather and food price changes.^(6)^	Consistently addressed the previous three pillars.

### Quality assessment

Four of the study investigators independently assessed the risk of bias in duplicate of the included studies using the ROBINS-I scale for non-randomised studies^(25)^ and ROB2^(26)^ for randomised studies. Any disagreements were resolved by the wider research team.

### Data analysis

Meta-analysis was considered as an analytical approach. However, due to the range of interventions and outcomes of interest, the data were heterogenous and therefore a meta-analysis was not possible. A narrative synthesis of the results was conducted, informed by the European Social Research Guidance on Conduct (ESRC) of Narrative Synthesis in Systematic Reviews.^(27)^ A framework comprising the four pillars of FI (acceptability, utilisation, accessibility, and stability) and intervention type was used to support synthesis of the results. Data synthesis was conducted by the primary investigator and discussed with the wider research team. The ESRC framework^(27)^ guided the narrative synthesis. The steps included developing a preliminary synthesis of findings, exploring relationships in the data and assessing the robustness of the synthesis. The recommended tools and techniques were assessed for suitability and studies were first organised by intervention type and the food security pillars addressed. Data were then tabulated into study summary and intervention contexts tables to develop a preliminary synthesis. The descriptive process evaluation data were mapped through idea webbing. Moderator variables were explored through tabulation and discussion of the intervention components and uptake data, to explore relationships in the data. To assess the robustness of the synthesis, quality assessments were conducted and tabulated. A reflection of the synthesis process is included in the limitations subsection of the discussion.

## Results

**Figure 1.**
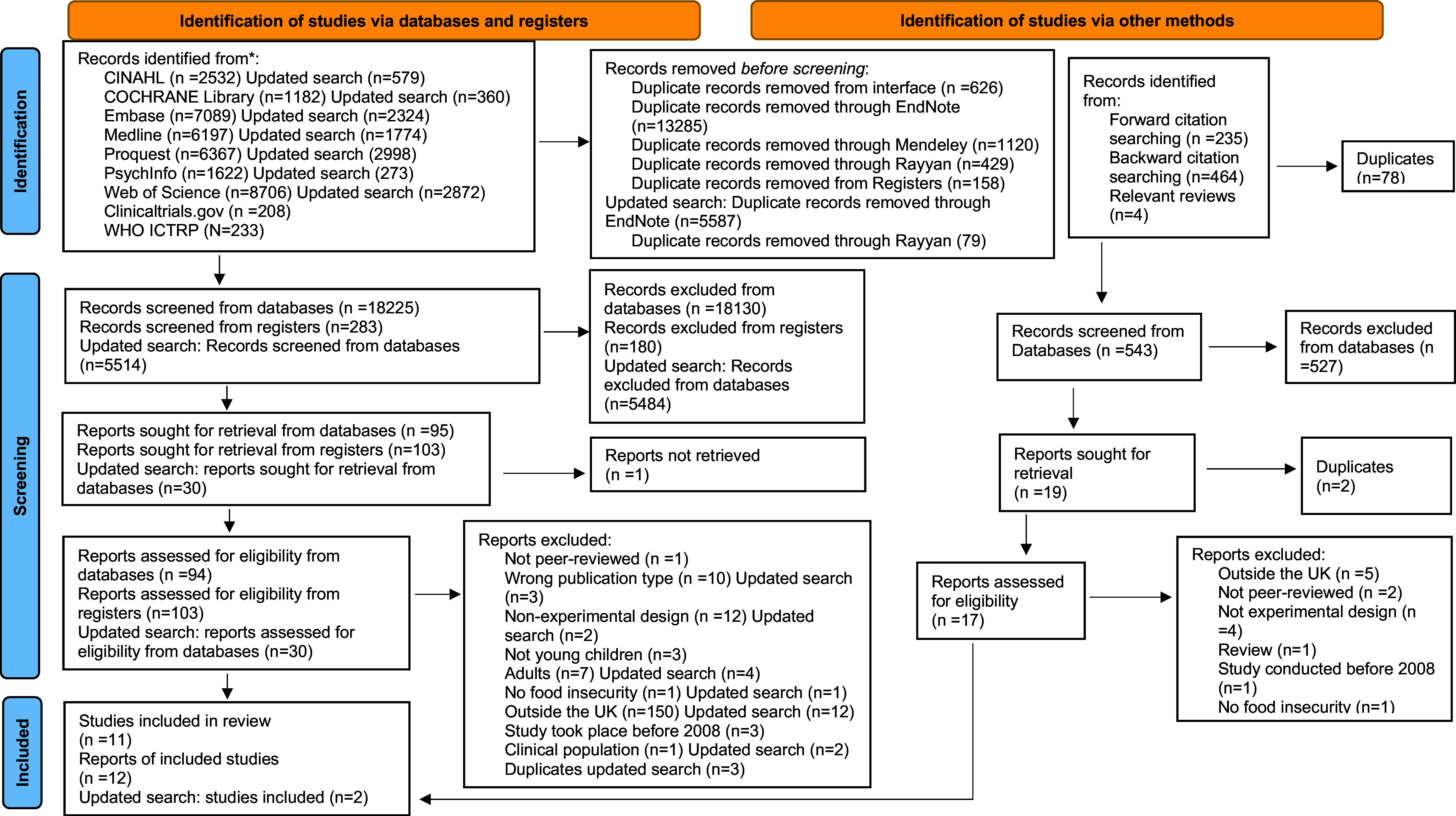
Figure 1 long description. PRISMA flow diagram.

Following deduplication, 18,225 (rapid search identified a further 5,514) articles were identified from the database searches, 283 from the trial registers searched, and 543 from citation chaining (Figure 1). 214 full texts were assessed in total across databases, registers, and through other methods. The rapid search identified a further 30 full texts which were assessed. A total of 11 intervention studies^(28–38)^ reported in 12 publications^(39)^ met the eligibility criteria and were included in this review. A further two intervention studies were identified in the rapid search^(40,41)^ that met the eligibility criteria. The 12 publications from the initial searches were analysed with a narrative synthesis with a narrative overview of the two interventions from the rapid searches provided.

Study characteristics:

Seven studies utilised a before-and-after design,^(28,31–34,37,38)^ two were quasi-experimental,^(29,35)^ one repeated cross-sectional ^(36)^ and one RCT^(30)^ (Table 3). Five studies took place in England,^(30,33,35,36,38)^ three in Scotland,^(32,34,37)^ two in Wales ^(28,29)^, and one across England and Scotland.^(31)^ Sample sizes ranged from 38 to 432 participants. Five of the reported interventions targeted children.^(29,31,35,36,38)^ Six targeted households,^(28,30,33,34,37)^ of which two targeted households with pregnant women.^(32,33)^ Studies were conducted between 2008 and 2021, the majority of which were conducted after 2015 (*n* = 7).^(28,29,31–35)^ Only one of the studies utilised a food security survey.^(28)^ The remaining studies utilised measures of income and deprivation to identify participants for recruitment (Table 4).

**Table 3.** Study characteristics Table 3 long description.

Authors, year	Location and data collection time period	Intervention			Study design	Participants		
		Setting	Components	Duration		Targeted population	Population size	Age range
Crilley et al. 2022 ^(35)^	London 2019	Holiday clubs in playgrounds and community centres	Children were served free lunches on holiday club days,	2 days (x2 24 hr recalls)	Quasi-experimental	Children	63	7–16 years
Garcia et al. 2014 ^(37)^	Ayrshire and Arran September 2010–January 2011	Varied venues	Cooking skills programme to raise awareness of the importance of healthy eating and to build up confidence and skills in cooking through practical, participatory sessions	4–8 weeks	Before and after	Parents of nursery-age children	102	17–45+ years
Garcia et al. 2017 ^(32)^	Glasgow, Inverclyde, Renfrewshire, East Renfrewshire January–February 2015	Community centres	Cooking classes to improve cooking confidence, skills and nutrition knowledge	6 weeks	Before and after	Pregnant women, households	117	16–45+ years
Garcia et al. 2019 ^(34)^	Glasgow and Clyde 2016–2017	Community centres	Cookery class with healthy eating education elements and practical activities	6 weeks	Before and after	Children	432	16–45+ years
Morgan et al. 2019 ^(29)^	Wales July–September 2016	Primary and secondary schools	Food and Fun: a multi-agency project providing healthy meals, nutrition skills and physical activities during the summer holiday period	6 weeks	Quasi-experimental	Children	196	3–14 years
Verfuerth et al. 2023 ^(28)^	Wales June–October 2021	Households	Weekly veg bags for 2–4 months from a community-supported agriculture farm over the harvest season	2 to 4 months	Before and after	Households	38	21–62 years
Parnham et al. 2022 ^(31)^	Across England and Scotland 2010–2017 and 2014–2017	Primary schools	Free school meals daily during the school days	4 years and 3 years	Before and after	Children	281	4–7 years
Spence et al. 2020 ^(36)^	Northeast England 2008–2009 and 2017–2019	Schools	Universal infant free school meals (a government scheme that provides all children between the ages of four and seven with free lunches in state funded schools)	2008–2009 and 2017–2018	Repeated cross-sectional survey	children	196	4–7 years
Thomas et al. 2022 ^(33)^	Yorkshire and Humber region February–August 2021	Supermarkets	Supermarket vouchers and top-up vouchers for low-income pregnant women and families with children under 4	6.5 months	Before and after	Households	133	18–65+ years
Watt et al. 2013 ^(30)^	Cornwall and Islington September 2010– November 2011	Children’s centres	2-hour sessions. Parents discussed and learned about a variety of aspects of healthy eating, second hour involved parents and children cook and eat session with basic food preparation and tasting	4 weeks	Randomised controlled trial	Families with children 18 months to five years	8 children’s centres	18 months to 5 years
Woodward et al. 2015 and Sahota et al. 2014 ^(38,39)^	Leeds January–October 2008	Primary schools and secondary schools	Intervention differed depending on each school, but areas focused on increasing free school meal claiming through; minimising discrimination, maximising awareness of free school meal.	Not reported	Before and after	Children	5 primary schools, 5 secondary schools	9–13 years

**Table 4.** Socio-economic status for included participants Table 4 long description.

	Socio-economic status	
Study	Measure of food insecurity/ deprivation	Socio-economic status of the sample
Crilley et al. 2022 ^(35)^	The English Indices of Deprivation	Holiday clubs located in areas of high deprivation, serving families normally in receipt of free school meals
Garcia et al. 2014 ^(37)^	Scottish Index of Multiple Deprivation (SIMD) quintiles	SIMD: Quintile 1 (38), 2 (36), 3 (9), 4 (10), 5 (5), Early Years Managers from each local authority identified target nurseries based on vulnerability of children and families.
Garcia et al. 2017 ^(32)^	Scottish Index of Multiple Deprivation (SIMD) quintiles	SIMD; quintile 1 (58.1%) 2 (17.7%), 3 (4.8%), 5 (4.8%)
Garcia et al. 2019 ^(34)^	Scottish Index of Multiple Deprivation (SIMD) quintiles	Scottish index of multiple deprivation quintile 1 (66%), 2 (14%), 3 (4%), 4 (4%), 5 (4%).
Morgan et al. 2019 ^(29)^	Child material deprivation index measure	Material deprivation (not deprived 53%, deprived 18.4%, very deprived 21.1%, severely deprived 7.5%)
Verfuerth et al. 2023 ^(28)^	Questions were adapted from USDA National Food Security Survey and United Kingdom median household income	High prevalence of experienced food insecurity, low income
Parnham et al. 2022 ^(31)^	Index of multiple deprivation (IMD) quintiles	IMD quintiles: least deprived (intervention group pre 19.1%, post 20.8%. Control group pre 19.4%, post 27.6%), 2 (intervention group pre 17.4%, post 17.7%. Control pre 14.9%, post 18.3%), 3 (intervention pre 23.2%, post 21%. Control pre 16.5%, post 13.5%) 4 (intervention pre 24%, post 19.1%. Control pre 21.2%, post 20.9%), most deprived (intervention pre 16.3%, post 21.4%. Control pre 28.1%, post 19.7%)
Spence et al. 2020 ^(36)^	The English Indices of Deprivation	School 1 was located in a more deprived ward and school 2 was in a less deprived ward
Thomas et al. 2022 ^(33)^	Lower Layer Super Output Area, corresponding for the index of multiple deprivation	Low income
Watt et al. 2013 ^(30)^	The English Indices of Deprivation	Took place in low-income communities
Woodward et al. 2015 ^(38,39)^	Free school meal (FSM) eligibility	High FSM eligibility

**Figure 2.**
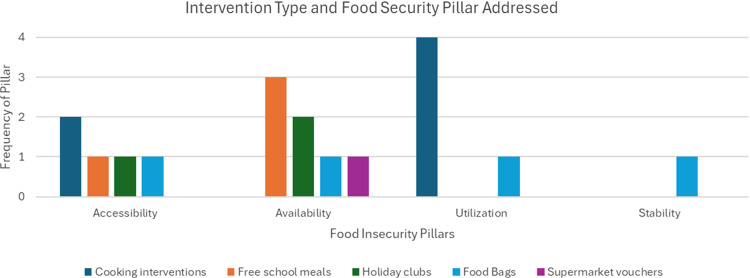
Figure 2 long description. Intervention and food security pillars addressed in each intervention (*n* = 11).

Seven studies were pilot interventions^(28–30,33,35,36)^, and four were full-scale interventions.^(31,32,34,37)^ The types of interventions varied (Figure 2): four utilised cooking classes,^(30,32,34,37)^ three free school meals (government scheme that provides children with free lunches if they meet the income related eligibility criteria),^(31,36,38)^ two holiday clubs,^(29,35)^ one supermarket vouchers,^(33)^ and one food bags (weekly vegetable bags from a community-supported agriculture farm).^(28)^ A theory of change was reported in only three studies. Watt et al.^(30)^ utilised two theories: social cognitive theory and theory of organisational change. Woodward et al. (2015) utilised a health-promoting schools model to inform the intervention, and Verfuerth et al. (2023) utilised the food well-being framework^(42)^ to inform their intervention’s design.

Four of the interventions took place in urban areas,^(32,34,35,38)^ five in a combination of rural and urban areas^(30,31,33,36,37)^ and two in rural areas.^(28,29)^ Intervention contexts (economic, seasonal, and local) also acted as mediating factors in three of the included studies ^(28,30,32)^ (Supplementary File 3).

There were 69 different outcomes reported. Outcomes relating to food consumption patterns were assessed in six of the studies,^(30–32,34,36,37)^ cooking practices were assessed in four ^(30,32,34,37)^ and nutrient intake, ^(31,36)^ food or nutrition knowledge, ^(32,37)^ and food cost were all assessed in two studies each.^(32,33)^ The remaining outcomes were assessed in one study each: confidence,^(37)^ school food standards,^(35)^ diet quality,^(35)^ food purchases,^(33)^ healthy eating opportunities,^(29)^ FI and wellbeing,^(28)^ and free school meals uptake ^(38)^. Outcomes were measured mostly through self-reported methods using questionnaires and interviews (*n* = 6).^(28,29,32,34,37,38)^ Five studies measured the weight (grams) or quantity of food/nutrients consumed,^(30,31,33,35,36)^ and one measured average spend on food (£).^(33)^ Only one study utilised a food security survey and directly measured FI as an outcome.^(28)^

We rated the risk of bias of seven of the non-randomised intervention studies as ‘serious’^(29,31–36)^ and three as ‘critical’ ^(28,37,38)^ (Table 5). The risk of bias assessment for the one RCT highlighted some concerns.^(30)^ Most studies (*n* = 10) were considered as having risk of bias for several reasons, such as covariates not being controlled for, missing data not addressed through appropriate analysis, and a lack of information to make an informed judgement.

**Table 5.**
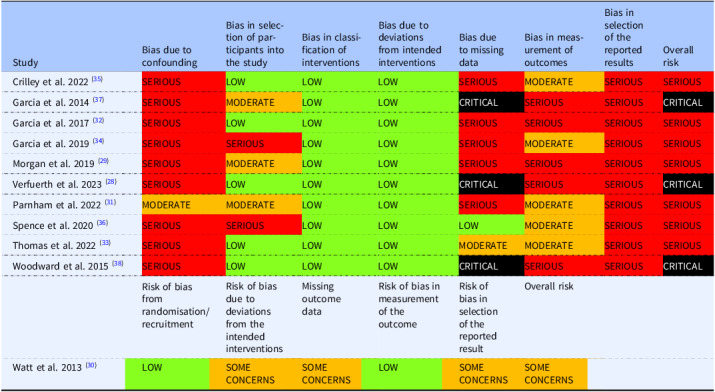
Risk of bias assessment results Table 5 long description.

### Synthesis of effectiveness results

For the food bags intervention, Verfuerth et al.^(28)^ found evidence for an increase in well-being scores among households (+0.29; 95% CI 0.46, 0.12; *p* < 0.05). For the food vouchers intervention, Thomas et al.^(33)^ reported 0.9 more portions of fruit and vegetables per day per household were purchased (*p* = 0.0017) and the percentage of fruit and vegetable weight within shopping baskets increased by 1.6% (*p* = 0.0242).

For the free school meal and holiday club interventions, Spence et al.^(36)^ found a decrease in pupils’ sugar intake (mean change –4.6%; 95% CI 6.3,−2.9; *p* = 0·77) following the introduction of free school meals. Parnham et al.^(31)^ found universal infant (4–7 years old) free school meals resulted in lower total fat (−2.5 g;−4.5,−0.5, 95% CI; *p* < 0.01), and Na intakes (−103.8mg;−163.1,−44.5, 95% CI; *p* < 0.01). However, there was no evidence it impacted energy or sugar intake. Morgan et al.^(29)^ found 86% of children reported consuming breakfast on holiday club days compared to 12% of children who did not eat breakfast at the club, as well as fewer sugary snacks (66%) and more fruit and vegetables (67%); however, no statistical tests were used. Crilley et al.^(35)^ reported evidence children’s diet quality improved on holiday club days (mean:58.0 SD12.6) versus non-club days (51.8 SD + 15.0; *p* = 0.007). Woodward et al.^(38)^ did not present intervention effectiveness data.

For the cooking education interventions, Garcia et al.^(37)^ reported evidence for increases in cooking confidence, and fruit and vegetable consumption among parents (*p* < 0.001). Garcia et al.^(32)^ reported improvements in the following confidence constructs: cooking using raw ingredients (*p* < 0.003), following a simple recipe (*p* = 0.003), shopping on a budget (*p* = 0.04), and cooking new foods (*p* < 0.001). Garcia et al.^(34)^ found families consumed less takeaway/fast foods (*p* = 0.019), ready meals (*p* = 0.003), and cooked more from scratch (*p* < 0.001). In addition, children’s consumption of sugary drinks (*p* = 0.012), biscuits (*p* = 0.007), and sweets/chocolates (*p* = 0.002) all reduced. Watt et al.^(30)^ did not present intervention effectiveness data.

### Pillar 1) Availability

Food availability was addressed in seven of the included studies^(28,29,31,33,35,36,38)^ (Figure 2)(Table 6). One of the cooking interventions^(37)^ partially accounted for availability by providing dishes for participants to take home; however, this was only one or two dishes weekly. All free school meal^(31,36,38)^ and holiday club^(29,35)^ interventions addressed availability by providing consistent food to the participants during the intervention. It was noted by the study authors that free school meal provisions provided to schools were not always sufficient to purchase an adequate amount of quality food for the children.^(31)^ The supermarket voucher intervention^(33)^ partially accounted for availability through top-up supermarket vouchers; however, participants reported this was often not a sufficient source of food and involved collecting the top-up voucher after completing their food shopping, therefore limiting its effectiveness. The food bags intervention^(28)^ provided consistent quality weekly vegetable bags to participants; however, participants reported having to supplement this to achieve full meals.

**Table 6.** Food security pillars addressed Table 6 long description.

	Food insecurity pillar targeted			
Study	Accessibility	Availability	Utilisation	Stability
Crilley et al. 2022 ^(35)^	No	Yes	No	No
Garcia et al. 2014 ^(37)^	Yes	No	Yes	No
Garcia et al. 2017 ^(32)^	Yes	No	Yes	No
Garcia et al. 2019 ^(34)^	No	No	Yes	No
Morgan et al. 2019 ^(29)^	Yes	Yes	No	No
Verfuerth et al. 2023 ^(28)^	Yes	Yes	Potentially utilisation (provided recipes and opportunities to visit the farms)	Potentially stability (although fluctuated depending on the length of the harvest season)
Parnham et al. 2022 ^(31)^	No	Yes	No	No
Spence et al. 2020 ^(36)^	No	Yes	No	No
Thomas et al. 2022 ^(33)^	No	Yes	No	No
Watt et al. 2013 ^(30)^	No	No	Yes	No
Woodward et al. 2015 ^(38,39)^	Yes	Yes	No	No

### Pillar 2) Accessibility

Food accessibility was addressed in five of the included studies. ^(28,29,32,37,38)^ Accessibility was addressed in cooking interventions, holiday clubs, free school meal, and food bag interventions. Two of the cooking interventions addressed accessibility,^(32,37)^ through incorporating elements of food planning and purchasing education, which may help participants to source food when shopping in supermarkets. The food bag intervention^(28)^ addressed accessibility as participants were provided with physical access to food delivered to their homes; however, participants reported this did not always meet their food preferences and did not account for economic accessibility, which may be necessary to supplement the vegetables provided.

### Pillar 3) Utilisation

Food utilisation was addressed in five of the included studies,^(28,30,32,34,37)^ using cooking-focused interventions and food bags. The food bag intervention addressed utilisation, through offering recipes and farm visits, although this was a supplementary aspect of the intervention. Three of the interventions were education-focused addressing nutritional knowledge and food practices through cooking classes, with healthy eating education and elements of practicalities of food planning and purchasing.^(32,34,37)^ Watt et al.^(30)^ provided nutrition, preparation, and cooking education, whilst also supplementing with mobile text updates to remind participants to continue to apply their learning.

### Pillar 4) Stability

Food stability was addressed in one of the included studies.^(28)^ In this study, weekly provision of food bags for participants was made feasible due to the food bags being sourced from local farms. However, the contents and consistency of quantity of the provided food fluctuated depending on the length of the harvest season. Only one study partially accounted for all the four pillars of FI.^(28)^

## Process evaluation

Drop-out rates were generally high across interventions, at post-intervention and follow-up measurements (Table 7). Where reported (*n* = 7), intervention uptake was predominantly above 60% (*n* = 5), with three studies achieving response rates above 80%.^(28,32,34)^

**Table 7.** Intervention components and uptake Table 7 long description.

Study	Intervention components	Response rate (percentage of those recruited from invited)	Attrition (number of participants who dropped out)	Duration	Frequency
Crilley et al. 2022 ^(35)^	Free lunches during weekdays at holiday programmes	Not reported, 63 participants recruited	84 participants in second time point (90%)	2 days (x2 24 hr recalls)	Daily lunches
Garcia et al. 2014 ^(37)^	Content varied depending on specific needs, wishes, and skills of the participants. Participants prepared and cooked one or more dishes to take home, a related nutrition activity or game on nutritional messages or info provided or shopping skills, for example, label reading and discussion of relevant supporting resources.	25.50%	356 (11%)	6 weeks	Weekly sessions lasting 2 hours
Garcia et al. 2017 ^(32)^	Elements of food planning, purchasing, preparation, cooking from scratch and storage. Practical cookery component and an educational element (healthy eating messages, activities).	86%	55 (53%)	6.5 months	Weekly vouchers
Garcia et al. 2019 ^(34)^	Weekly cooking classes with healthy eating education elements and practical activities e.g. how to read food labels, understanding food traffic light system, tips to achieve 5 a day fruit and veg recommendations and prepare healthy meals	83.70%	374 (13%)	2 days (x2 24 hr recalls)	Club day and non-club day
Morgan et al. 2019 ^(29)^	Two healthy meals a day and physical activities	60.70%	Not reported	6 weeks	12 days over 6 weeks
Verfuerth et al. 2023 ^(28)^	Weekly veg bags, recipes, farm visits	86.40%	22 (42%)	2 to 4 months	Weekly
Parnham et al. 2022 ^(31)^	Universal infant free school meals at lunch	75.80%	Not reported	4 years + 3 years	Free school meals daily during the school days
Spence et al. 2020 ^(36)^	Free school meals	Not reported, 196 participants recruited	58 (75%)	4 to 8 weeks	Weekly sessions lasting 2 hours
Thomas et al. 2022 ^(33)^	Weekly supermarket top-up vouchers of £2	9.60%	Not reported	6 weeks	6 × 2-hour sessions
Watt et al. 2013 ^(30)^	Nutrition teaching, how to cook and prepare basic foods, tasting sessions, CHERRY at home to remind participants to continue their learnings	Not reported, 394 participants recruited	90 (77%)	4 weeks	Weekly, 4
Woodward et al. 2015 ^(38,39)^	Varied across schools, to minimise discrimination (designated FSM contact, amending anti-bullying policies, assemblies, lessons on nutrition), to maximise FSM awareness (postcards and letters to all parents eligible, in school posters, school events, newsletters, websites, texts, letters to parents not taking up their entitlement).	Not reported, 5 primary and 5 secondary schools recruited	Not reported	NA	During school days

## Barriers

**Figure 3.**
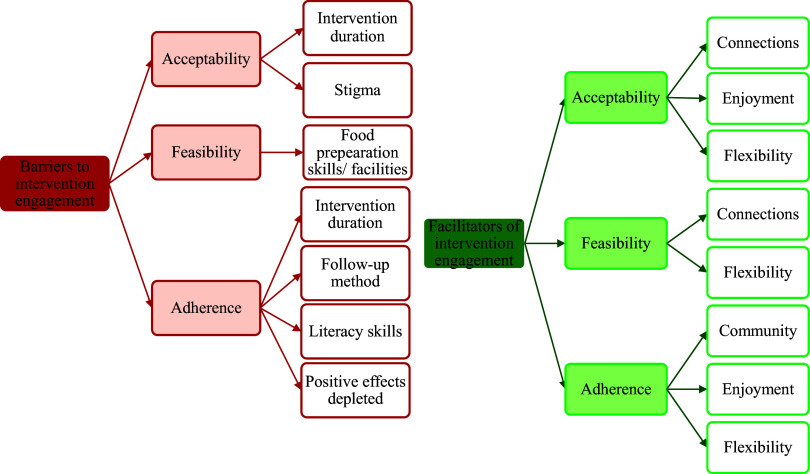
Figure 3 long description. Facilitators and barriers to acceptability, feasibility, and adherence to intervention engagement.

Stigma was an issue in several of the studies (Figure 3), particularly for free school meal provision interventions and supermarket vouchers. Participants reported feeling unable to engage with the interventions due to the questionnaire methods used, which presented a barrier for those with limited literacy skills.^(32)^ As such, flexibility through visual communication was suggested by study authors to improve engagement.^(39)^ Follow-up methods were consistently insufficient to maintain adherence as noted by study authors.^(34,37)^ Furthermore, positive intervention effects consistently reduced at follow-up. In the case of learning-based interventions, authors noted refresher sessions may support maintenance.^(37)^

## Facilitators

Features that increased participant acceptability included flexibility (Figure 3), such as the timing of the intervention sessions being suitable for participants, a sense of independence and choice for participants’ food selections, cultural appropriateness of the intervention components and having transport available for participants where necessary. Existing connections that were utilised in the designing and planning of the interventions improved these interventions’ feasibility and acceptability to participants. These included utilising existing structures and local organisations the participants may already attend and trust. Enjoyment for participants was also key for acceptability and adherence; approaches that were preferred by participants included having activities for children, utilising a whole-family approach for household-targeted interventions and encouraging participants to try new foods.

Community was an important feature that encouraged adherence. Incorporating a social element into interventions increased enjoyment and engagement for participants. Finally, flexible approaches were beneficial to adherence, for example, providing transportation allowed participants to continue to attend the intervention. Additionally, where relevant, providing child support for caregiver-focused interventions also facilitated adherence as reported by study authors.^(34)^

Two further intervention studies were identified in the rapid search that met the eligibility criteria. The first was a quasi-experimental holiday club intervention conducted in a local authority in the West Midlands. The study focused on caregivers’ views on the Holiday Activities and Food (HAF) programme during 2021 and 2022. The HAF programme provides free food and activities for children aged 5–16 years old in receipt of means-tested free school meals during the school holidays.^(40)^ The second study was a natural experiment of a free school meals intervention conducted in four local authorities in Greater London. The study focused on primary schools that received universal free school meals in 2009/10 to 2014/15, with the sample comprising year 6 pupils aged 10–11 years. Both studies included a programme group and a control group.^(41)^

The holiday club intervention reported no significant differences for household FI or perceptions of child safety. For children who attended for 6 weeks or more, parents were found to be more concerned about affordable childcare and less stressed than those who did not attend the programme. For levels of physical activity, they found that children who attended for more than three weeks engaged in more physical activity.^(40)^ The free school meals intervention found no significant effects of the scheme during the initial six years on attainment or school absences. However, improvements were observed for reading and mathematics attainment for children who had experienced UFSM throughout their primary school years in two of the schools that had implemented the scheme for the longest.^(41)^

Both intervention studies addressed two pillars of food security, availability, and accessibility, which are in line with the other intervention studies in this review that were free school meals and holiday clubs. This supports our findings that there is a lack of intervention studies addressing all pillars of food security. Both studies measured outcomes that differed from those reported in other studies in this review, and Defeyter et al. (40) also measured food security, as in the food bag provision intervention,^(28)^ although this was not found to be impacted by the intervention.

## Discussion

This review explored UK interventions that addressed the pillars of FI for children, families with children and pregnant women, post-2008. We identified 11 interventions that addressed at least one of the four pillars of food security. We found there is a lack of high-quality rigorous intervention studies undertaken in the UK, with only one study explicitly including FI as an outcome, and only one study partially accounting for all food security pillars in its intervention design.^(4) (28)^ Consistent with other reviews,^(18,20)^ the scope and quality of interventions that addressed FI was limited. Flexibility, participant enjoyment, utilising existing connections and a communal atmosphere were all found to be important features of feasible and acceptable interventions. Whereas stigma, intervention duration, and high literacy demands of participants were reported as barriers to intervention success.

One of the objectives of this review was to assess which food security pillars were addressed in UK interventions. Most studies evaluated existing UK government policies, including the Healthy Start scheme; through the provision of additional top-up supermarket vouchers,^(33)^ holiday clubs,^(29,35)^ and free school meals.^(31,36,38)^ This review found most interventions did not effectively address all the necessary pillars to alleviate FI and predominantly targeted availability, whilst failing to address the remaining pillars. Whilst food availability was the focus of the majority of the included interventions, the interventions did not completely fulfil participants’ need for food availability, with interventions often being unable to provide an adequate amount of food.^(31,33,38)^ In order to be able to address the pillars of food security fully, additional funding and support is necessary; however, this remains a challenge in the current UK economic climate.^(43)^ Therefore, a holistic approach, such as innovative improvements to increase the availability and affordability of food, such as urban agriculture initiatives^(44)^ and strategies that aim to address the root social determinants of FI, such as a affordable housing programmes, improving transportation infrastructure and promoting liveable wages and income inequality are needed.^(45,46)^

Previous UK research highlighted school breakfast provision positively impacts the school environment by providing a calm and positive start to the day for children, whilst reducing children’s short-term hunger.^(5)^ Cohen et al.^(22)^ further supports these findings, demonstrating free school meals may be associated with improved household income and food security. However, prior research highlights consistent issues with these approaches, for example, Gatenby^(47)^ demonstrates many children in receipt of free school meals were not consuming adequate portions of food in schools. This is similar to the findings of this review, which demonstrate participants and providers believe school food standards need to improve and the current free school meal allowance for schools is insufficient. Holiday clubs were seen as acceptable by participants and, as reported by previous research,^(48)^ can alleviate FI for at-risk populations during school holidays. Similar issues were apparent in holiday clubs, which often had insufficient organisation and food availability due to a lack of funding.^(29)^ Providing interventions through schools was considered feasible and acceptable for participants. This has the potential to support food availability and accessibility at least partially if further support and funding is provided.

One study explored the use of supermarket vouchers (as a supplement to the vouchers provided by the Healthy Start scheme) and found uptake was low due to insufficient provider funds and a complicated system for receiving the vouchers. Previous research reports that^(5)^ Healthy Start vouchers do not fully cover recipients’ average weekly spend on fruit and vegetables and are not associated with an increase in fruit and vegetable expenditure.^(31)^ The value of the voucher limits food availability and economic accessibility, as participants are required to supplement the funds to purchase a sufficient amount of food. As a more accessible alternative, research suggests the provision of cash transfers allows individuals the independence to choose to spend the money on what they need most,^(49)^ to both reduce stigma and allow for agency. Appropriate nutritional education may also be required to supplement cash transfers to ensure sufficient nutrition literacy.

One study utilised free food bags. Consistent with previous research, this intervention was viewed as acceptable to participants,^(50)^ whilst partially addressing the pillars of food security. Whilst food utilisation was addressed through recipes and farm visits, this may not be feasible or suitable for participants to engage with if participants do not have the tools or facilities required to cook or the time and transport to visit the farm. Further research is needed to understand how this type of intervention could be implemented on a wider scale, whilst focusing on reducing the issue of harvest seasons affecting how long food provisions are available to participants and thus impacting food stability. However, none of the included interventions sufficiently addressed utilisation within the home environment, thus, we were not able to assess whether participants had the facilities or means to cook at home and apply the intervention-based learning. These findings indicate combining food provisions with other methods, such as providing basic cookware, may be beneficial.

The findings suggest stigma was a barrier to intervention acceptability and adherence, particularly in school-based interventions. Previous research suggested holiday clubs face issues with social acceptability, inaccessibility, and unreliability.^(5)^ Therefore, universal non-means-tested policies or discretion may be suitable strategies to adopt to reduce the impacts of stigma. However, this may impact the resources needed to facilitate these clubs. The location of the interventions was an important mediating factor in this review and previous research.^(51)^ Utilising existing locations such as schools and community centres increased acceptability for participants and reduced barriers to adherence.^(51)^

Optimal intervention duration was not possible to establish between the included studies, suggesting this may vary depending on the type of intervention and participants’ needs. McKay et al.^(19)^ found longer interventions with early intervention were key to success. As discussed in this review, a range of features are preferred by participants,^(19)^ such as a whole-family approach with multiple activities. Charlton et al.^(52)^ found nutrition education programmes, including food provision, cooking classes, and school gardens, were most successful if they included multiple strategies, and had parental involvement and take-home activities for the children.

Across the included studies it was found utilising existing community facilities or groups for the design, recruitment, and implementation of the intervention benefitted feasibility and acceptability for both participants and providers. Previous research highlighted the benefits of utilising local community members to offer a variety of activities to children and families in holiday clubs.^(51)^ Therefore, community-led interventions may be a way to ensure interventions are culturally appropriate and feasible. A review exploring interventions in developed countries to tackle children’s FI reported that the social benefits of holiday clubs increased participant uptake.^(18)^ This review demonstrated that families valued a communal and social element to the intervention, which increased enjoyment, and reduced stigma.

Flexibility was a common facilitator of intervention acceptability. Important elements of flexibility included timing of the intervention, participant choice in the selection of food, and transport. Whilst these elements varied depending on the type of intervention, location, and target population, it is nevertheless important to consider the most acceptable and feasible approach, by discussing these elements with the participants in the design of the intervention.

Finally, some methodological concerns consistently featured. Self-report methods were used for data collection by most of the studies. This presented issues, as participants frequently did not complete questionnaires or completed them incorrectly. Therefore, 24-hour recalls and food diaries were recommended by some of the authors^(30,32)^; however, literacy skills would need to be carefully considered. The majority of the included studies assessed income or geographical deprivation as a proxy for FI, which may not accurately capture all experiences of FI. FI was only measured directly by one study, demonstrating how infrequently FI is measured in the UK in peer-reviewed literature. Furthermore, most of the interventions were small-scale pilots, limiting the interpretation of effectiveness across the studies and the quality of the studies.

## Strengths and limitations

This review adopted a rigorous systematic methodology and an extensive search strategy. It is the first review to explore what interventions that addressed FI targeted at children, families, and pregnant women have been conducted in the UK. However, this review had some limitations. Firstly, due to the limited UK-based literature in this field, the inclusion criteria remained broad. Therefore, data were heterogeneous, and a meta-analysis was not possible. This limited our ability to draw firm conclusions about intervention effectiveness. It is possible the criteria we used to identify interventions that addressed FI included some studies which were not intending to address FI specifically. Furthermore, by focusing on experimental studies, alternative evaluations of existing UK schemes that did not utilise an experimental design were excluded, such as studies evaluating the Healthy Start Scheme. Additionally, by excluding grey literature, further relevant interventions may have been excluded.

There was a lack of studies including preschool-aged children (0–5 years old) and pregnant women, and further research is needed with these populations to understand the different challenges, which should be accounted for in designing and implementing FI-focused policies and interventions in these groups. Furthermore, research should include cost-effectiveness evaluations.

## Conclusion

This review identified a lack of high-quality interventions addressing all of the pillars of FI for children, families with children, and pregnant women in the UK. There was a lack of interventions targeting pregnant women and young children (0–5 year). Interventions which targeted households utilised cooking interventions and nutrition education, whilst interventions which targeted children mainly utilised free school meals and holiday clubs. A range of barriers (e.g. stigma, literacy skills, food preparation skills) and facilitators (e.g. flexibility, community engagement, and enjoyment) were identified to inform future intervention development. To design interventions that can fully address the pillars of food security, a holistic approach is needed to address the root social determinants of FI, through further policies and funding at government levels and by utilising existing structures that people are already accessing. Further research is needed in this area to explore how interventions that address FI could be implemented, in a way that is acceptable to participants and feasible for providers, through co-production with families experiencing FI to address the barriers and facilitators identified in this review.

## Supporting information

10.1017/jns.2026.10111.sm001 Holt et al. supplementary material 1 Holt et al. supplementary material

10.1017/jns.2026.10111.sm002 Holt et al. supplementary material 2 Holt et al. supplementary material

10.1017/jns.2026.10111.sm003 Holt et al. supplementary material 3 Holt et al. supplementary material

## Supplementary material

The supplementary material for this article can be found at https://doi.org/10.1017/jns.2026.10111.

## Table 1. Long description

The table presents the eligibility criteria for a study, structured according to the PICOS framework. It includes five columns: Population, Intervention, Comparison, Outcomes, and Study design. The Population column specifies that the majority of participants must be children aged 0-11 years old, families with at least one child aged 0-11 years, or pregnant women. The Intervention column indicates that the study was conducted in the UK and addresses at least one of the pillars of FI (accessibility, utilization, availability, or stability). The Comparison column states that no eligibility criteria were applied relating to comparison groups. The Outcomes column mentions that no eligibility criteria were applied relating to reported outcomes. The Study design column includes randomized controlled trials, before-and-after studies, quasi-experimental studies, factorial design plots, studies, and feasibility studies, or qualitative studies of an intervention with an experimental design. The table provides a detailed breakdown of the criteria used to determine the eligibility of participants and studies for inclusion.

Navigate back to Table 1..

## Table 2. Long description

The table presents a comparison of food security pillars, their definitions, and the types of characteristics used to assess which pillars are addressed in each intervention. It includes four main columns: Food Insecurity, Definition, and Intervention Characteristics. The Food Insecurity column lists different aspects of food insecurity such as Availability, Accessibility, Utilization, and Stability. The Definition column provides detailed descriptions of each aspect. The Intervention Characteristics column outlines specific criteria used to evaluate the interventions. The table is structured with multiple rows, each detailing the specific definitions and characteristics for each pillar. Notable trends include detailed criteria for assessing the effectiveness of interventions in addressing food insecurity.

Navigate back to Table 2..

## Figure 1. Long description

The flowchart begins with the identification of studies via databases and registers, listing sources such as CINAHL, COCHRANE Library, Embase, Medline, Proquest, PsycINFO, Web of Science, Clinicaltrials.gov, and WHO ICTRP. It details the number of records identified and updated searches. Duplicates are removed before screening, with specific numbers for each removal method. Records are then screened from databases and registers, with exclusions noted. Reports are sought for retrieval, and further exclusions are made based on criteria such as peer review status, publication type, experimental design, age group, and relevance to the UK. The process includes an updated search with similar steps. Studies are included in the review based on eligibility assessments. The flowchart also shows the identification of studies via other methods, including forward and backward citation searching and relevant reviews, with duplicates and exclusions noted. The final included studies and reports are summarized.

Navigate back to Figure 1..

## Table 3. Long description

The table presents a detailed summary of study characteristics, focusing on the design, location, sample sizes, and target populations of various interventions. It includes seven studies that utilized a before-and-after design, two quasi-experimental studies, one repeated cross-sectional study, and one randomized controlled trial. The studies took place in different regions of the United Kingdom, with five in England, three in Scotland, two in Wales, and one across England and Scotland. Sample sizes ranged from 38 to 432 participants. Five of the interventions targeted children, while six targeted households, with two specifically targeting households with pregnant women. The studies were conducted between 2008 and 2021, with the majority conducted after 2015. Only one study utilized a food security survey, while the others used measures of income and deprivation for participant recruitment.

Navigate back to Table 3..

## Table 4. Long description

The table presents socio-economic status data for participants included in various studies. It lists studies by author and year, detailing socio-economic status, socio-economic status of the sample, and notes. The table includes data from studies conducted in different regions, with sample sizes ranging from 38 to 432 participants. Five studies targeted children, six targeted households, and two specifically targeted households with pregnant women. Studies were conducted between 2008 and 2021, with the majority conducted after 2015. The table also notes the use of food security surveys and measures of income and deprivation for participant recruitment.

Navigate back to Table 4..

## Figure 2. Long description

The bar graph titled Intervention Type and Food Security Pillar Addressed compares the frequency of different food insecurity pillars addressed by various interventions. The x-axis represents the food insecurity pillars: Accessibility, Availability, Utilization, and Stability. The y-axis represents the frequency of each pillar. The graph includes five data series represented by different colors: Cooking interventions in dark blue, Free school meals in orange, Holiday clubs in green, Food Bags in light blue, and Supermarket vouchers in purple. For Accessibility, Cooking interventions have a frequency of 2, while the other interventions each have a frequency of 1. For Availability, Free school meals have a frequency of 3, Holiday clubs have a frequency of 2, and the other interventions each have a frequency of 1. For Utilization, Cooking interventions have a frequency of 4, Food Bags have a frequency of 1, and the other interventions have a frequency of 0. For Stability, Food Bags have a frequency of 1, and the other interventions have a frequency of 0. All values are approximated.

Navigate back to Figure 2..

## Table 5. Long description

The table presents a risk of bias assessment for multiple studies, focusing on areas such as bias due to confounding, selection of participants, classification of interventions, deviations from intended interventions, missing data, measurement of outcomes, and selection of reported results. The table includes 11 rows and 8 columns, with each row representing a different study and each column representing a specific bias category. Notable studies include those by Crilley et al. 2022, Garcia et al. 2014, and Gantt et al. 2017, among others. The risk levels range from low to critical, with most studies showing serious risks in several categories. For instance, Crilley et al. 2022 has serious risk due to confounding and missing data but moderate risk in measurement of outcomes. Garcia et al. 2014 shows critical risk due to deviations from intended interventions. The table provides a comprehensive overview of the bias risks associated with each study, aiding in the evaluation of their reliability and validity.

Navigate back to Table 5..

## Table 6. Long description

The table presents a summary of various studies on food insecurity pillars, specifically targeting accessibility, availability, utilization, and stability. It includes data from multiple studies, such as Crilley et al. 2022, Garcia et al. 2014, Garcia et al. 2017, Garcia et al. 2019, Morgan et al. 2019, Verfueth et al. 2023, Parnham et al. 2022, Spence et al. 2020, Thomas et al. 2022, Watt et al. 2013, and Woodward et al. 2015. The table is organized into columns labeled Study, Accessibility, Availability, Utilisation, and Stability. Each row corresponds to a different study and indicates whether the study addressed each pillar with ‘Yes’ or ‘No’. Notable trends include the consistent addressing of availability in studies like Garcia et al. 2014, Garcia et al. 2017, and Morgan et al. 2019, while utilization is less frequently addressed. Stability is not addressed in any of the studies. The table provides a clear comparison of how different studies have targeted various aspects of food insecurity.

Navigate back to Table 6..

## Table 7. Long description

The table presents a comparison of intervention components and their uptake across different studies. It includes columns for study identifiers, intervention components, response rates, attrition, duration, and frequency. The table has 15 rows and 7 columns, detailing specific interventions, their design, and participant response rates. Notable trends include high drop-out rates and predominantly high uptake rates above 60 percent in most studies.

Navigate back to Table 7..

## Figure 3. Long description

The diagram presents a structured overview of the facilitators and barriers to intervention engagement, divided into three main categories: acceptability, feasibility, and adherence. On the left side, barriers to intervention engagement are listed under each category. For acceptability, the barriers include intervention duration and stigma. For feasibility, the barriers are food preparation skills or facilities and intervention duration. For adherence, the barriers are follow-up method, literacy skills, and positive effects depleted. On the right side, facilitators of intervention engagement are listed under the same categories. For acceptability, the facilitators are connections, enjoyment, and flexibility. For feasibility, the facilitators are connections, flexibility, and community. For adherence, the facilitators are enjoyment and flexibility. Arrows indicate the flow from barriers to facilitators, showing how addressing barriers can lead to enhanced engagement.

Navigate back to Figure 3..
