# Supplementary material for: Interventions that address food insecurity for children aged 0–11 years, families, and pregnant women in the UK: a systematic review of intervention studies
Source: J Nutr Sci. 2026 Jun 11;15:e42. doi: 10.1017/jns.2026.10111 (PMC13279956; doi:10.1017/jns.2026.10111)
Supplement: Holt et al. supplementary material 1 — Holt et al. supplementary material [file S2048679026101116sup001.docx]

**Supplementary File 1: Search Strategies for Databases**

|  | **Ovid MEDLINE(R) Search Strategy, Date searched: 25/10/2023** |
| --- | --- |
|  | **Concept 1: food insecurity** |
| 1 | food supply/ or famine/ or food deserts/ or food insecurity/ or access to healthy foods/ or food security/ |
| 2 | "food poverty".mp. |
| 3 | "food insufficiency".mp. |
| 4 | "food hardship".mp. |
| 5 | Food Assistance/ |
| 6 | "lack of food".mp. |
| 7 | "food rationing".mp. |
| 8 | "nutrition security".mp. |
| 9 | relief work/ or food assistance/ |
| 10 | "food availability".mp. |
| 11 | Food Deprivation/ |
| 12 | "food shortage".mp. |
| 13 | "food deficient".mp. |
| 14 | "food scarcity".mp. |
| 15 | "food prescription".mp. |
| 16 | "produce prescription".mp. |
| 17 | "food stress".mp. |
| 18 | "food stability".mp. |
| 19 | "food instability".mp. |
| 20 | "food reserve".mp. |
| 21 | "community kitchen".mp. |
| 22 | "food program".mp. |
| 23 | "community food".mp. |
| 24 | "market voucher".mp. |
| 25 | "food availability".mp. |
| 26 | "food inaccessibility".mp. |
| 27 | "food unavailability".mp. |
| 28 | "food accessibility".mp. |
| 29 | "food inaccessibility".mp. |
| 30 | "food utilization".mp. |
| 31 | "food stability".mp. |
| 32 | "food instability".mp. |
| 33 | "food steadiness".mp. |
| 34 | "food supply".mp. |
| 35 | famine.mp. |
| 36 | "food deserts".mp. |
| 37 | "food insecurity".mp. |
| 38 | "access to healthy foods".mp. |
| 39 | "food security".mp. |
| 40 | "food deprivation".mp. |
| 41 | 1 or 2 or 3 or 4 or 5 or 6 or 7 or 8 or 9 or 10 or 11 or 12 or 13 or 14 or 15 or 16 or 17 or 18 or 19 or 20 or 21 or 22 or 23 or 24 or 25 or 26 or 27 or 28 or 29 or 30 or 31 or 32 or 33 or 34 or 35 or 36 or 37 or 38 or 39 or 40 |
|  | **Concept 2a: children** |
| 42 | child/ or child, preschool/ or infant/ or minors/ |
| 43 | toddler.mp. |

| 44 | Infant, Newborn/ |
| --- | --- |
| 45 | kids.mp. |
| 46 | Pediatrics/ |
| 47 | "early years".mp. |
| 48 | Child Day Care Centers/ or Child, Preschool/ or Child Care/ |
| 49 | boy*.mp. |
| 50 | girl*.mp. |
| 51 | juvenil*.mp. |
| 52 | schools/ or schools, nursery/ |
| 53 | schoolchild.mp. |
| 54 | classroom.mp. |
| 55 | youth.mp. |
| 56 | Child.mp. |
| 57 | Preschool.mp. |
| 58 | Infants.mp. |
| 59 | Minors.mp. |
| 60 | Children.mp. |
| 61 | Newborns.mp. |
| 62 | Pediatric*.mp. |
| 63 | "child care".mp. |
| 64 | Childcare.mp. |
| 65 | "child day care centers".mp. |
| 66 | Nursery.mp. |
| 67 | School*.mp. |
| 68 | Nurseries.mp. |
| 69 | 42 or 43 or 44 or 45 or 46 or 47 or 48 or 49 or 50 or 51 or 52 or 53 or 54 or 55 or 56 or 57 or  58 or 59 or 60 or 61 or 62 or 63 or 64 or 65 or 66 or 67 or 68 |
|  | **Concept 2b: families** |
| 70 | family/ or parents/ |
| 71 | fathers/ or mothers/ |
| 72 | carer*.mp. |
| 73 | caregiver*.mp. |
| 74 | Family.mp. |
| 75 | Parent*.mp. |
| 76 | Father*.mp. |
| 77 | Mother*.mp. |
| 78 | 70 or 71 or 72 or 73 or 74 or 75 or 76 or 77 |
|  | **Concept 2c: pregnant women** |
| 79 | Pregnant Women/ |
| 80 | maternal.mp. |
| 81 | preconception.mp. |
| 82 | prenatal.mp. |
| 83 | Pregnancy/ |
| 84 | antenatal.mp. |
| 85 | Postpartum Period/ |
| 86 | postnatal.mp. |
| 87 | gestation.mp. |
| 88 | Parturition/ |
| 89 | "pregnant women".mp. |
| 90 | "pregnant woman".mp. |

| 91 | Pregnancy.mp. |
| --- | --- |
| 92 | Postpartum.mp. |
| 93 | Parturition.mp. |
| 94 | 79 or 80 or 81 or 82 or 83 or 84 or 85 or 86 or 87 or 88 or 89 or 90 or 91 or 92 or 93 |
| 95 | 69 or 78 or 94 |
| 96 | 41 and 95 |
|  | **Concept 3: interventions** |
| 97 | intervention.mp. |
| 98 | program*.mp. |
| 99 | activit*.mp. |
| 100 | government programs/ or policy/ |
| 101 | guideline/ |
| 102 | controlled clinical trials as topic/ or non-randomized controlled trials as topic/ or randomized controlled trials as topic/ or feasibility studies/ or pilot projects/ |
| 103 | control groups/ or random allocation/ |
| 104 | "before and after".mp. |
| 105 | pretest.mp. |
| 106 | "pre test".mp. |
| 107 | posttest.mp. |
| 108 | "post test".mp. |
| 109 | "time series".mp. |
| 110 | "time point".mp. |
| 111 | "at random".mp. |
| 112 | "repeated measure".mp. |
| 113 | controlled before-after studies/ or interrupted time series analysis/ |
| 114 | "attention control".mp. |
| 115 | trial.mp. |
| 116 | compar*.mp. |
| 117 | "random* assign".mp. |
| 118 | randomisation.mp. |
| 119 | Random Allocation/ |
| 120 | RCT.mp. |
| 121 | trial.mp. |
| 122 | factorial.mp. |
| 123 | pre-intervention.mp. |
| 124 | post-intervention.mp. |
| 125 | quasi*.mp. |
| 126 | "natural experiment*".mp. |
| 127 | "government program*".mp. |
| 128 | Policy.mp. |
| 129 | Guideline.mp. |
| 130 | "controlled clinical trial*".mp. |
| 131 | "non-randomized controlled trial*".mp. |
| 132 | "randomized controlled trial*".mp. |
| 133 | "feasibility study".mp. |
| 134 | "pilot project".mp. |
| 135 | "control group*".mp. |
| 136 | "random allocation".mp. |
| 137 | "controlled before-after studies".mp. |
| 138 | "interrupted time series analysis".mp. |
| 139 | "random allocation".mp. |
| 140 | "nested qualitative".mp. |
| 141 | "qualitative nested".mp. |
| 142 | (qualitative adj3 experimental).mp. |
| 143 | (qualitative adj3 "feasibility study").mp. |
| 144 | 97 or 98 or 99 or 100 or 101 or 102 or 103 or 104 or 105 or 106 or 107 or 108 or 109 or 110 or 111 or 112 or 113 or 114 or 115 or 115 or 116 or 117 or 117 or 118 or 119 or 120 or 121 or 122 or 123 or 124 or 125 or 126 or 127 or 128 or 129 or 130 or 131 or 132 or 133 or 134 or 135 or 136 or 137 or 138 or 139 or 140 or 141 or 142 or 143 |
| 145 | 96 and 144 |
| 146 | limit 145 to yr="2008 -Current" 6,184 results |

| **Web of science: 8706 results, Date searched: 25/10/2023** |
| --- |
| ((TS=("food assistance" OR "food poverty" "food insufficiency" OR "food hardship" OR "lack of food" OR "food rationing" OR "nutrition security" OR "food availability" OR "food deprivation" OR "food shortage" OR "food deficient" OR "food scarcity" OR "food prescription" OR "produce prescription" OR "food stress" OR "food stability" OR "food instability" OR "food reserve" OR "community kitchen" OR "market voucher" OR "food program" OR "community food" OR "food availability" OR "food inaccessibility" OR "food unavailability" OR "food acceptability" OR "food inaccessibility" OR "food utili?ation" OR "food stability" OR "food instability" OR "food steadiness" OR "food supply" OR "food instability" OR "food steadiness" OR "food supply" OR "food deserts" OR "food insecurity" OR "access to healthy food*" OR "food security" OR "food deprivation" OR "free school meal*" OR "breakfast provision*" OR "food provision*" OR "lunch provision*" OR "free breakfast*")) AND TS=(toddler* OR kids OR "early years" OR boy* OR girl* OR juvenil* OR schoolchild OR schoolchildren* OR classroom* OR youth OR child* OR preschool* OR infant* OR minor* OR children* OR newborn* OR pediatric* OR "child care" OR childcare OR "child day care" OR nursery OR nurseries OR school* OR carer* OR caregiver* OR family OR families OR father* OR mother* OR maternal OR preconception OR prenatal OR antenatal OR postnatal OR gestation OR pregnant OR pregnancy OR postpartum OR parturition)) AND TS=("pilot study" OR intervention* OR "before and after" OR pretest OR "pre test" OR posttest OR "post test" OR "time series" OR "time point" OR "at random" OR "repeated meaure*" OR "attention control" OR trial* OR compar* OR "random* assign*" OR randomi?ation OR RCT OR factorial OR pre-intervention OR post-intervention OR quasi* OR "natural experiment*" OR "controlled clinical trial*" OR "non-randomi?ed controlled trial*" OR "randomi?ed controlled trial*" OR "feasibility study" OR "pilot project" OR "control group*" OR "random allocation" OR "controlled before-after stud*" OR "interrupted time series analysis" OR "nested qualitative" OR "qualitative nested" OR "process evaluation" OR program* OR qualitative NEAR/3 experimental OR qualitative NEAR/3 "feasibility study") |

| **APA PsycInfo, Date searched: 25/10/2023** | **1622 results** |
| --- | --- |
| 1 | "food supply".mp. |
| 2 | food deprivation/ or food insecurity/ |
| 3 | "food desert*".mp. |
| 4 | "food insecurity".mp. |
| 5 | "food security".mp. |
| 6 | "food assistance".mp. |
| 7 | "food poverty".mp. |
| 8 | "food insufficiency".mp. |
| 9 | "food hardship".mp. |
| 10 | "lack of food".mp. |
| 11 | "food rationing".mp. |
| 12 | "nutrition security".mp. |
| 13 | "food availability".mp. |
| 14 | "nutrition insecurity".mp. |
| 15 | "food deprivation".mp. |
| 16 | "food shortage".mp. |
| 17 | "food deficient".mp. |
| 18 | "food scarcity".mp. |
| 19 | "food prescription".mp. |
| 20 | "produce prescription".mp. |
| 21 | "food stress".mp. |
| 22 | "food stability".mp. |
| 23 | "food instability".mp. |
| 24 | "food reserve".mp. |
| 25 | "community kitchen".mp. |
| 26 | "market voucher".mp. |
| 27 | "food program".mp. |
| 28 | "community food".mp. |
| 29 | "food availability".mp. |
| 30 | "food inaccessibility".mp. |
| 31 | "food unavailability".mp. |
| 32 | "food acceptability".mp. |
| 33 | "food inaccessibility".mp. |
| 34 | "food utilisation".mp. |
| 35 | "food utilization".mp. |
| 36 | "food stability".mp. |
| 37 | "food instability".mp. |
| 38 | "food steadiness".mp. |
| 39 | "access to healthy food*".mp. |
| 40 | "free school meal*".mp. |
| 41 | "breakfast provision*".mp. |
| 42 | "food provision*".mp. |
| 43 | "free breakfast*".mp. |
| 44 | 1 or 2 or 3 or 4 or 5 or 6 or 7 or 8 or 9 or 10 or 11 or 12 or 13 or 14 or 15 or 16 or 17 or 18 or 19 or 20 or 21 or 22 or 23 or 24 or 25 or 26 or 27 or 28 or 29 or 30 or 31 or 32 or 33 or 34 or 35 or 36 or 37 or 38 or 39 or 40 or 41 or 42 or 43 |
| 45 | child.mp. |
| 46 | preschool students/ or nursery school students/ or kindergarten students/ |
| 47 | preschool*.mp. |
| 48 | infant*.mp. |
| 49 | minor*.mp. |
| 50 | toddler*.mp. |
| 51 | newborn*.mp. |
| 52 | kids.mp. |
| 53 | pediatrics/ |
| 54 | pediatric*.mp. |
| 55 | "early years".mp. |
| 56 | child day care/ or day care centers/ |
| 57 | "child day care".mp. |
| 58 | nursery schools/ |
| 59 | nurseries.mp. |
| 60 | boys.mp. |
| 61 | girls.mp. |
| 62 | juvenil*.mp. |
| 63 | child care/ |
| 64 | "child care".mp. |
| 65 | school*.mp. |
| 66 | schoolchild*.mp. |
| 67 | classroom*.mp. |
| 68 | youth.mp. |
| 69 | nursery.mp. |
| 70 | primary school students/ |
| 71 | 45 or 46 or 47 or 48 or 49 or 50 or 51 or 52 or 53 or 54 or 55 or 56 or 57 or 58 or 59 or 60 or 61 or 62 or 63 or 64 or 65 or 66 or 67 or 68 or 69 or 70 |
| 72 | family/ |
| 73 | "family".mp. |
| 74 | families.mp. |
| 75 | parent*.mp. |
| 76 | father*.mp. |
| 77 | mother*.mp. |
| 78 | carer*.mp. |
| 79 | caregivers/ |
| 80 | caregiver*.mp. |
| 81 | 72 or 73 or 74 or 75 or 76 or 77 or 78 or 79 or 80 |
| 82 | pregnancy/ or perinatal period/ or postnatal period/ or prenatal care/ |
| 83 | pregnant.mp. |
| 84 | pregnancy.mp. |
| 85 | maternal.mp. |
| 86 | preconception.mp. |
| 87 | prenatal.mp. |
| 88 | parturition.mp. |
| 89 | antenatal.mp. |
| 90 | postpartum.mp. |
| 91 | postnatal.mp. |
| 92 | gestation.mp. |
| 93 | 82 or 83 or 84 or 85 or 86 or 87 or 88 or 89 or 90 or 91 or 92 |
| 94 | 71 or 81 or 93 |
| 95 | "pilot study".mp. |
| 96 | intervention/ or early intervention/ or family intervention/ or school based intervention/ |
| 97 | intervention.mp. |
| 98 | "controlled clinical trial".mp. |
| 99 | "randomised controlled trial".mp. |
| 100 | "randomized controlled trial".mp. |
| 101 | randomized controlled trials/ or randomized clinical trials/ or experimental design/ |
| 102 | "non-randomized controlled trial".mp. |
| 103 | "non-randomised controlled trial".mp. |
| 104 | "feasibility study".mp. |
| 105 | "pilot project".mp. |
| 106 | "random allocation".mp. |
| 107 | "control group*".mp. |
| 108 | "before and after".mp. |
| 109 | pretest.mp. |
| 110 | "pre test".mp. |
| 111 | posttest.mp. |
| 112 | "post test".mp. |
| 113 | "time series".mp. |
| 114 | "time point".mp. |
| 115 | "at random".mp. |
| 116 | repeated measures/ or posttesting/ or pretesting/ |
| 117 | experimental design/ or quasi experimental methods/ |
| 118 | "repeated measure*".mp. |
| 119 | time series/ |
| 120 | "controlled before-after study".mp. |
| 121 | "interrupted time series analysis".mp. |
| 122 | "attention control".mp. |
| 123 | trial.mp. |
| 124 | compar*.mp. |
| 125 | "random* assign*".mp. |
| 126 | randomisation.mp. |
| 127 | randomization.mp. |
| 128 | "random allocation".mp. |
| 129 | RCT.mp. |
| 130 | factorial.mp. |
| 131 | pre-intervention.mp. |
| 132 | post-intervention.mp. |
| 133 | quasi*.mp. |
| 134 | "natural experiment*".mp. |
| 135 | "nested qualitative".mp. |
| 136 | "qualitative nested".mp. |
| 137 | "process evaluation".mp. |
| 138 | program*.mp. |
| 139 | (qualitative adj3 experimental).mp. [mp=title, abstract, heading word, table of contents, key concepts, original title, tests & measures, mesh word] |
| 140 | (qualitative adj3 "feasibility study").mp. [mp=title, abstract, heading word, table of contents, key concepts, original title, tests & measures, mesh word] |
| 141 | 95 or 96 or 97 or 98 or 99 or 100 or 101 or 102 or 103 or 104 or 105 or 106 or 107 or 108 or 109 or 110 or 111 or 112 or 113 or 114 or 115 or 116 or 117 or 118 or 119 or 120 or 121 or 122 or 123 or 124 or 125 or 126 or 127 or 128 or 129 or 130 or 131 or 132 or 133 or 134 or 135 or 136 or 137 or 138 or 139 or 140 |
| 142 | 44 and 94 and 141 |
| 143 | limit 142 to yr="2008 -Current" |

| **Embase, Date searched: 25/10/2023** | **7089 results** |
| --- | --- |
| 1 | "food supply".mp. |
| 2 | food desert/ or food insecurity/ |
| 3 | "food deserts".mp. |
| 4 | "food insecurity".mp. |
| 5 | food security/ |
| 6 | "food security".mp. |
| 7 | food assistance/ or food availability/ |
| 8 | "food assistance".mp. |
| 9 | "food poverty".mp. |
| 10 | "food insufficiency".mp. |
| 11 | "food hardship".mp. |
| 12 | "lack of food".mp. |
| 13 | "food rationing".mp. |
| 14 | "nutrition security".mp. |
| 15 | "nutrition insecurity".mp. |
| 16 | "food availability".mp. |
| 17 | food deprivation/ |
| 18 | "food deprivation".mp. |
| 19 | "food shortage".mp. |
| 20 | "food deficient".mp. |
| 21 | "food scarcity".mp. |
| 22 | "food prescription".mp. |
| 23 | "produce prescription".mp. |
| 24 | "food stress".mp. |
| 25 | "food stability".mp. |
| 26 | "food instability".mp. |
| 27 | "food reserve".mp. |
| 28 | "community kitchen".mp. |
| 29 | "market voucher".mp. |
| 30 | "food program".mp. |
| 31 | "community food".mp. |
| 32 | "food availability".mp. |
| 33 | "food inaccessibility".mp. |
| 34 | "food unavailability".mp. |
| 35 | "food acceptability".mp. |
| 36 | "food utilisation".mp. |
| 37 | "food utilization".mp. |
| 38 | "food stability".mp. |
| 39 | "food instability".mp. |
| 40 | "food steadiness".mp. |
| 41 | "access to healthy food*".mp. |
| 42 | "free school meal*".mp. |
| 43 | "breakfast provision*".mp. |
| 44 | "food provision*".mp. |
| 45 | "lunch provision*".mp. |
| 46 | "free breakfast*".mp. |
| 47 | 1 or 2 or 3 or 4 or 5 or 6 or 7 or 8 or 9 or 10 or 11 or 12 or 13 or 14 or 15 or 16 or 17 or 18 or 19 or 20 or 21 or 22 or 23 or 24 or 25 or 26 or 27 or 28 or 29 or 30 or 31 or 32 or 33 or 34 or 35 or 36 or 37 or 38 or 39 or 40 or 41 or 42 or 43 or 44 or 45 or 46 |
| 48 | child/ or juvenile/ or boy/ or girl/ or infant/ or preschool child/ or school child/ or toddler/ |
| 49 | preschool.mp. |
| 50 | infant/ or baby/ or newborn/ |
| 51 | infant*.mp. |
| 52 | minors.mp. |
| 53 | "minor (person)"/ |
| 54 | toddler*.mp. |
| 55 | newborn*.mp. |
| 56 | pediatric*.mp. |
| 57 | "early years".mp. |
| 58 | child day care/ |
| 59 | "child day care".mp. |
| 60 | nursery/ or nursery school/ |
| 61 | nursery.mp. |
| 62 | nurseries.mp. |
| 63 | child care/ or kindergarten/ or nursery/ |
| 64 | "child care".mp. |
| 65 | boys.mp. |
| 66 | girls.mp. |
| 67 | juvenil*.mp. |
| 68 | primary school/ or school/ |
| 69 | school*.mp. |
| 70 | school child/ |
| 71 | schoolchild.mp. |
| 72 | classroom.mp. |
| 73 | schoolchildren*.mp. |
| 74 | youth.mp. |
| 75 | child*.mp. |
| 76 | child*.mp. |
| 77 | childcare.mp. |
| 78 | 48 or 49 or 50 or 51 or 52 or 53 or 54 or 55 or 56 or 57 or 58 or 59 or 60 or 61 or 62 or 63 or 64 or 65 or 66 or 67 or 68 or 69 or 70 or 71 or 72 or 73 or 74 or 75 or 76 or 77 |
| 79 | family/ |
| 80 | family.mp. |
| 81 | families.mp. |
| 82 | parent/ or father/ or mother/ |
| 83 | parent*.mp. |
| 84 | father*.mp. |
| 85 | mother*.mp. |
| 86 | carer*.mp. |
| 87 | caregiver/ |
| 88 | caregiver*.mp. |
| 89 | 79 or 80 or 81 or 82 or 83 or 84 or 85 or 86 or 87 or 88 |
| 90 | maternal.mp. |
| 91 | pregnant woman/ |
| 92 | pregnant.mp. |
| 93 | preconception.mp. |
| 94 | prenatal.mp. |
| 95 | pregnancy/ |
| 96 | pregnancy.mp. |
| 97 | parturition.mp. |
| 98 | antenatal.mp. |
| 99 | postpartum.mp. |
| 100 | gestation.mp. |
| 101 | 90 or 91 or 92 or 93 or 94 or 95 or 96 or 97 or 98 or 99 or 100 |
| 102 | 78 or 89 or 101 |
| 103 | pilot study/ |
| 104 | "pilot study".mp. |
| 105 | intervention study/ |
| 106 | intervention.mp. |
| 107 | controlled clinical trial/ or randomized controlled trial/ or "controlled clinical trial (topic)"/ |
| 108 | "controlled clinical trial".mp. |
| 109 | "randomised controlled trial".mp. |
| 110 | "randomized controlled trial".mp. |
| 111 | randomization/ or randomized controlled trial/ |
| 112 | "non-randomized controlled trial".mp. |
| 113 | "non-randomised controlled trial".mp. |
| 114 | feasibility study/ |
| 115 | "feasibility study".mp. |
| 116 | "pilot project".mp. |
| 117 | "control group*".mp. |
| 118 | "random allocation".mp. |
| 119 | "before and after".mp. |
| 120 | pretest.mp. |
| 121 | "pre test".mp. |
| 122 | posttest.mp. |
| 123 | "post test".mp. |
| 124 | "time series".mp. |
| 125 | "time point".mp. |
| 126 | time series analysis/ |
| 127 | "at random".mp. |
| 128 | "repeated measure*".mp. |
| 129 | "controlled before-after study".mp. |
| 130 | "interrupted time series analysis".mp. |
| 131 | "attention control".mp. |
| 132 | trial.mp. |
| 133 | compar*.mp. |
| 134 | compar*.mp. |
| 135 | "random* assign*".mp. |
| 136 | randomisation.mp. |
| 137 | randomization.mp. |
| 138 | "random allocation".mp. |
| 139 | RCT.mp. |
| 140 | factorial.mp. |
| 141 | pre-intervention.mp. |
| 142 | post-intervention.mp. |
| 143 | quasi*.mp. |
| 144 | "natural experiment".mp. |
| 145 | "nested qualitative".mp. |
| 146 | "qualitative nested".mp. |
| 147 | "process evaluation".mp. |
| 148 | program.mp. |
| 149 | (qualitative adj3 experimental).mp. [mp=title, abstract, heading word, drug trade name, original title, device manufacturer, drug manufacturer, device trade name, keyword heading word, floating subheading word, candidate term word] |
| 150 | (qualitative adj3 "feasibility study").mp. [mp=title, abstract, heading word, drug trade name, original title, device manufacturer, drug manufacturer, device trade name, keyword heading word, floating subheading word, candidate term word] |
| 151 | 103 or 104 or 105 or 106 or 107 or 108 or 109 or 110 or 111 or 112 or 113 or 114 or 115 or 116 or 117 or 118 or 119 or 120 or 121 or 122 or 123 or 124 or 125 or 126 or 127 or 128 or 129 or 130 or 131 or 132 or 133 or 134 or 135 or 136 or 137 or 138 or 139 or 140 or 141 or 142 or 143 or 144 or 145 or 146 or 147 or 148 or 149 or 150 |
| 152 | 47 and 102 and 151 |
| 153 | limit 152 to yr="2008 -Current" |

| **Proquest: 6367 results, Date searched: 25/10/2023** |
| --- |
| noft("food assistance" OR "food poverty" "food insufficiency" OR "food hardship" OR "lack of food" OR "food rationing" OR "nutrition security" OR "food availability" OR "food deprivation" OR "food shortage" OR "food deficient" OR "food scarcity" OR "food prescription" OR "produce prescription" OR "food stress" OR "food stability" OR "food instability" OR "food reserve" OR "community kitchen" OR "market voucher" OR "food program" OR "community food" OR "food availability" OR "food inaccessibility" OR "food unavailability" OR "food acceptability" OR "food inaccessibility" OR "food utili?ation" OR "food stability" OR "food instability" OR "food steadiness" OR "food supply" OR "food instability" OR "food steadiness" OR "food supply" OR "food deserts" OR "food insecurity" OR "access to healthy food*" OR "food security" OR "food deprivation" OR "free school meal*" OR "breakfast provision*" OR "food provision*" OR "lunch provision*" OR "free breakfast*") AND noft(toddler* OR kids OR "early years" OR boy* OR girl* OR juvenil* OR schoolchild OR schoolchildren* OR classroom* OR youth OR child* OR preschool* OR infant* OR minor* OR children* OR newborn* OR pediatric* OR "child care" OR childcare OR "child day care" OR nursery OR nurseries OR school* OR carer* OR caregiver* OR family OR families OR father* OR mother* OR maternal OR preconception OR prenatal OR antenatal OR postnatal OR gestation OR pregnant OR pregnancy OR postpartum OR parturition) AND noft("pilot study" OR intervention* OR "before and after" OR pretest OR "pre test" OR posttest OR "post test" OR "time series" OR "time point" OR "at random" OR "repeated meaure*" OR "attention control" OR trial* OR compar* OR "random* assign*" OR randomi?ation OR RCT OR factorial OR pre-intervention OR post-intervention OR quasi* OR "natural experiment*" OR "controlled clinical trial*" OR "non-randomi?ed controlled trial*" OR "randomi?ed controlled trial*" OR "feasibility study" OR "pilot project" OR "control group*" OR "random allocation" OR "controlled before-after stud*" OR "interrupted time series analysis" OR "nested qualitative" OR "qualitative nested" OR "process evaluation" OR program* OR qualitative NEAR/3 experimental OR qualitative NEAR/3 "feasibility study") |

| **CINAHL: 2532 results, Date searched: 25/10/2023** |
| --- |
| "food assistance" OR "food poverty" "food insufficiency" OR "food hardship" OR "lack of food" OR "food rationing" OR "nutrition security" OR "food availability" OR "food deprivation" OR "food shortage" OR "food deficient" OR "food scarcity" OR "food prescription" OR "produce prescription" OR "food stress" OR "food stability" OR "food instability" OR "food reserve" OR "community kitchen" OR "market voucher" OR "food program" OR "community food" OR "food availability" OR "food inaccessibility" OR "food unavailability" OR "food acceptability" OR "food inaccessibility" OR "food utili?ation" OR "food stability" OR "food instability" OR "food steadiness" OR "food supply" OR "food instability" OR "food steadiness" OR "food supply" OR "food deserts" OR "food insecurity" OR "access to healthy food*" OR "food security" OR "food deprivation" OR "free school meal*" OR "breakfast provision*" OR "food provision*" OR "lunch provision*" OR "free breakfast*" |
| AND |
| toddler* OR kids OR "early years" OR boy* OR girl* OR juvenil* OR schoolchild OR schoolchildren* OR classroom* OR youth OR child* OR preschool* OR infant* OR minor* OR children* OR newborn* OR pediatric* OR "child care" OR childcare OR "child day care" OR nursery OR nurseries OR school* OR carer* OR caregiver* OR family OR families OR father* OR mother* OR maternal OR preconception OR prenatal OR antenatal OR postnatal OR gestation OR pregnant OR pregnancy OR postpartum OR parturition |
| AND |
| "pilot study" OR intervention* OR "before and after" OR pretest OR "pre test" OR posttest OR "post test" OR "time series" OR "time point" OR "at random" OR "repeated meaure*" OR "attention control" OR trial* OR compar* OR "random* assign*" OR randomi?ation OR RCT OR factorial OR pre-intervention OR post-intervention OR quasi* OR "natural experiment*" OR "controlled clinical trial*" OR "non-randomi?ed controlled trial*" OR "randomi?ed controlled trial*" OR "feasibility study" OR "pilot project" OR "control group*" OR "random allocation" OR "controlled before-after stud*" OR "interrupted time series analysis" OR "nested qualitative" OR "qualitative nested" OR "process evaluation" OR program* OR qualitative NEAR3 experimental OR qualitative NEAR3 "feasibility study" |

| **COCHRANE library: 1182 results** | **Concept, Date searched: 25/10/2023** |
| --- | --- |
| ID | Search |
| #1 | ("food supply"):ti,ab,kw (Word variations have been searched) |
| #2 | MeSH descriptor: [Food Supply] this term only |
| #3 | ("food deserts"):ti,ab,kw (Word variations have been searched) |
| #4 | MeSH descriptor: [Food Deserts] this term only |
| #5 | ("food insecurity"):ti,ab,kw (Word variations have been searched) |
| #6 | MeSH descriptor: [Food Insecurity] this term only |
| #7 | ("food security"):ti,ab,kw (Word variations have been searched) |
| #8 | MeSH descriptor: [Food Security] this term only |
| #9 | ("food assistance"):ti,ab,kw (Word variations have been searched) |
| #10 | MeSH descriptor: [Food Assistance] this term only |
| #11 | ("food poverty"):ti,ab,kw (Word variations have been searched) |
| #12 | ("food insufficiency"):ti,ab,kw (Word variations have been searched) |
| #13 | ("food hardship"):ti,ab,kw (Word variations have been searched) |
| #14 | ("lack of food"):ti,ab,kw (Word variations have been searched) |
| #15 | ("food rationing"):ti,ab,kw (Word variations have been searched) |
| #16 | ("nutrition security"):ti,ab,kw (Word variations have been searched) |
| #17 | ("nutrition insecurity"):ti,ab,kw (Word variations have been searched) |
| #18 | ("food availability"):ti,ab,kw (Word variations have been searched) |
| #19 | MeSH descriptor: [Access to Healthy Foods] this term only |
| #20 | ("food deprivation"):ti,ab,kw (Word variations have been searched) |
| #21 | MeSH descriptor: [Food Deprivation] this term only |
| #22 | ("food shortage"):ti,ab,kw (Word variations have been searched) |
| #23 | ("food deficient"):ti,ab,kw (Word variations have been searched) |
| #24 | ("food scarcity"):ti,ab,kw (Word variations have been searched) |
| #25 | ("food prescription"):ti,ab,kw (Word variations have been searched) |
| #26 | ("produce prescription"):ti,ab,kw (Word variations have been searched) |
| #27 | ("food stress"):ti,ab,kw (Word variations have been searched) |
| #28 | ("food stability"):ti,ab,kw (Word variations have been searched) |
| #29 | ("food instability"):ti,ab,kw (Word variations have been searched) |
| #30 | ("food reserve"):ti,ab,kw (Word variations have been searched) |
| #31 | ("community kitchen"):ti,ab,kw (Word variations have been searched) |
| #32 | ("market voucher"):ti,ab,kw (Word variations have been searched) |
| #33 | ("food program"):ti,ab,kw (Word variations have been searched) |
| #34 | ("community food"):ti,ab,kw (Word variations have been searched) |
| #35 | ("food availability"):ti,ab,kw (Word variations have been searched) |
| #36 | ("food inaccessibility"):ti,ab,kw (Word variations have been searched) |
| #37 | ("food unavailability"):ti,ab,kw (Word variations have been searched) |
| #38 | ("food acceptability"):ti,ab,kw (Word variations have been searched) |
| #39 | ("food stability"):ti,ab,kw (Word variations have been searched) |
| #40 | ("food instability"):ti,ab,kw (Word variations have been searched) |
| #41 | ("food steadiness"):ti,ab,kw (Word variations have been searched) |
| #42 | (food NEXT utili?ation):ti,ab,kw (Word variations have been searched) |
| #43 | ("access to healthy food"):ti,ab,kw (Word variations have been searched) |
| #44 | ("access to healthy foods"):ti,ab,kw (Word variations have been searched) |
| #45 | ("free school meals"):ti,ab,kw (Word variations have been searched) |
| #46 | ("free school meal"):ti,ab,kw (Word variations have been searched) |
| #47 | ("breakfast provision"):ti,ab,kw (Word variations have been searched) |
| #48 | ("food provision"):ti,ab,kw (Word variations have been searched) |
| #49 | ("lunch provision"):ti,ab,kw (Word variations have been searched) |
| #50 | ("free breakfast"):ti,ab,kw (Word variations have been searched) |
| #51 | ("free breakfasts"):ti,ab,kw (Word variations have been searched) |
| #52 | #1 OR #2 OR #3 OR #4 OR #5 OR #6 OR #7 OR #8 OR #9 OR #10 OR #11 OR #12 OR #13 OR #14 OR #15 OR #16 OR #17 OR #18 OR #19 OR #20 OR #21 OR #22 OR #23 OR #24 OR #25 OR #26 OR #27 OR #28 OR #29 OR #30 OR #31 OR #32 OR #33 OR #34 OR #35 OR #36 OR #37 OR #38 OR #39 OR #40 OR #41 OR #42 OR #43 OR #44 OR #45 OR #46 OR #47 OR #48 OR #49 OR #50 OR #51 |
| #53 | MeSH descriptor: [Child] this term only |
| #54 | MeSH descriptor: [Infant] explode all trees |
| #55 | (preschool*):ti,ab,kw (Word variations have been searched) |
| #56 | MeSH descriptor: [Infant, Newborn] this term only |
| #57 | MeSH descriptor: [Minors] this term only |
| #58 | MeSH descriptor: [Child Day Care Centers] explode all trees |
| #59 | MeSH descriptor: [Schools, Nursery] this term only |
| #60 | MeSH descriptor: [Child Care] this term only |
| #61 | MeSH descriptor: [Schools] this term only |
| #62 | (infant*):ti,ab,kw (Word variations have been searched) |
| #63 | (minors):ti,ab,kw (Word variations have been searched) |
| #64 | (toddler*):ti,ab,kw (Word variations have been searched) |
| #65 | (newborn*):ti,ab,kw (Word variations have been searched) |
| #66 | (pediatric*):ti,ab,kw (Word variations have been searched) |
| #67 | ("early years"):ti,ab,kw (Word variations have been searched) |
| #68 | ("child day care"):ti,ab,kw (Word variations have been searched) |
| #69 | (nursery):ti,ab,kw (Word variations have been searched) |
| #70 | (nurseries):ti,ab,kw (Word variations have been searched) |
| #71 | ("child care"):ti,ab,kw (Word variations have been searched) |
| #72 | (boys):ti,ab,kw (Word variations have been searched) |
| #73 | (girls):ti,ab,kw (Word variations have been searched) |
| #74 | (juvenil*):ti,ab,kw (Word variations have been searched) |
| #75 | (school*):ti,ab,kw (Word variations have been searched) |
| #76 | (schoolchild):ti,ab,kw (Word variations have been searched) |
| #77 | (classroom):ti,ab,kw (Word variations have been searched) |
| #78 | (schoolchildren*):ti,ab,kw (Word variations have been searched) |
| #79 | (youth):ti,ab,kw (Word variations have been searched) |
| #80 | (child*):ti,ab,kw (Word variations have been searched) |
| #81 | (childcare):ti,ab,kw (Word variations have been searched) |
| #82 | #53 OR #54 OR #55 OR #56 OR #57 OR #58 OR #59 OR #60 OR #61 OR #62 OR #63 OR #64 OR #65 OR #66 OR #67 OR #68 OR #69 OR #70 OR #71 OR #72 OR #73 OR #74 OR #75 OR #76 OR #77 OR #78 OR #79 OR #80 OR #81 |
| #83 | MeSH descriptor: [Family] this term only |
| #84 | MeSH descriptor: [Parents] this term only |
| #85 | MeSH descriptor: [Fathers] this term only |
| #86 | MeSH descriptor: [Mothers] this term only |
| #87 | MeSH descriptor: [Caregivers] this term only |
| #88 | (family):ti,ab,kw (Word variations have been searched) |
| #89 | (families):ti,ab,kw (Word variations have been searched) |
| #90 | (parent*):ti,ab,kw (Word variations have been searched) |
| #91 | (father*):ti,ab,kw (Word variations have been searched) |
| #92 | (mother*):ti,ab,kw (Word variations have been searched) |
| #93 | (carer*):ti,ab,kw (Word variations have been searched) |
| #94 | (caregiver*):ti,ab,kw (Word variations have been searched) |
| #95 | #83 OR #84 OR #85 OR #86 OR #87 OR #88 OR #89 OR #90 OR #91 OR #92 OR #93 OR #94 |
| #96 | MeSH descriptor: [Pregnant Women] this term only |
| #97 | MeSH descriptor: [Pregnancy] this term only |
| #98 | (maternal):ti,ab,kw (Word variations have been searched) |
| #99 | (pregnant):ti,ab,kw (Word variations have been searched) |
| #100 | (preconception):ti,ab,kw (Word variations have been searched) |
| #101 | (prenatal):ti,ab,kw (Word variations have been searched) |
| #102 | (pregnancy):ti,ab,kw (Word variations have been searched) |
| #103 | (parturition):ti,ab,kw (Word variations have been searched) |
| #104 | (antenatal):ti,ab,kw (Word variations have been searched) |
| #105 | (postpartum):ti,ab,kw (Word variations have been searched) |
| #106 | (gestation):ti,ab,kw (Word variations have been searched) |
| #107 | #96 OR #97 OR #98 OR #99 OR #100 OR #101 OR #102 OR #103 OR #104 OR #105 OR #106 |
| #108 | #82 OR #95 OR #107 |
| #109 | #52 AND #108 with Publication Year from 2008 to 2023, with Cochrane Library publication date Between Jan 2008 and Sep 2023, in Trials |
