# Supplementary material for: Interventions that address food insecurity for children aged 0–11 years, families, and pregnant women in the UK: a systematic review of intervention studies
Source: J Nutr Sci. 2026 Jun 11;15:e42. doi: 10.1017/jns.2026.10111 (PMC13279956; doi:10.1017/jns.2026.10111)
Supplement: Holt et al. supplementary material 2 — Holt et al. supplementary material [file S2048679026101116sup002.docx]

| **Clinicaltrials.gov:** |
| --- |
| Condition or disease: |
| "Food Insecurity" OR "Food Supply" OR "food deserts" OR "access to healthy foods" OR "Food Security" OR "food poverty" OR "food insufficiency" OR "food hardship" OR "food assistance" OR "lack of food" OR "food rationing" OR "nutrition security" OR "nutrition insecurity" OR "food availability" OR "food deprivation" OR "food shortage" OR "food deficient" OR “food scarcity" OR "food stress" OR "food stability" OR "food instability" OR "community kitchen" OR "food program" OR "food availability" OR "food inaccessibility" OR "food unavailability" OR "food accessibility" OR "food inaccessibility" OR "food utilisation" OR "food stability" OR "food instability" OR "food steadiness" |
| 208 results |
| Date searched: 19/12/2023 |

**Supplementary File 2: Trial Register Search Strategies**

| **WHO ICTRP:** |
| --- |
| "Food Insecurity" OR "Food Supply" OR "food deserts" OR "access to healthy foods" OR "Food Security" OR "food poverty" OR "food insufficiency" OR "food hardship" OR "food assistance" OR "lack of food" OR "food rationing" OR "nutrition security" OR "nutrition insecurity" OR "food availability" OR "food deprivation" OR "food shortage" OR "food deficient" OR “food scarcity" OR "food stress" OR "food stability" OR "food instability" OR "community kitchen" OR "food program" OR "food availability" OR "food inaccessibility" OR "food unavailability" OR "food accessibility" OR "food inaccessibility" OR "food utilisation" OR "food stability" OR "food instability" OR "food steadiness" |
| 233 results |
| Date searched: 19/12/2023 |
