# Supplementary material for: Interventions that address food insecurity for children aged 0–11 years, families, and pregnant women in the UK: a systematic review of intervention studies
Source: J Nutr Sci. 2026 Jun 11;15:e42. doi: 10.1017/jns.2026.10111 (PMC13279956; doi:10.1017/jns.2026.10111)
Supplement: Holt et al. supplementary material 3 — Holt et al. supplementary material [file S2048679026101116sup003.docx]

| **Supplementary File 3: Table of Intervention Contexts** | | | | | |
| --- | --- | --- | --- | --- | --- |
| **Study** | **UK Country** | **Specific Location** | **Geographical Context** | **Setting** | **Intervention Context** |
| Crilley et al 2022 ^(34)^ | England | London | Urban | Varied venues | NA |
| Garcia et al 2014 ^(36)^ | Scotland | Ayrshire and Arran | Mixed | Varied venues | NA |
| Garcia et al 2017 ^(31)^ | Scotland | Glasgow, Ivernclyde, Renfrewshire and East Refrewshire | Urban | Community centres | Economic context- Shifts in purchasing during the pandemic |
| Garcia et al 2019 ^(33)^ | Scotland | Glasgow and Clyde | Urban | Schools | NA |
| Morgan et al 2019 ^(28)^ | Wales | Wales | Rural | Primary and secondary schools | NA |
| Verfuerth et al 2023 ^(27)^ | Wales | Wales | Rural | Households | Seasonal context- contents of food bags differed due to seasonality of produce. |
| Parnham et al 2022 ^(30)^ | England and Scotland | Across England and Scotland | Mixed | Primary schools | NA |
| Spence et al 2020 ^(35)^ | England | Northeast England | Mixed | Schools | NA |
| Thomas et al 2022 ^(32)^ | England | Yorkshire and Humber region | Mixed | Holiday clubs and community centres | NA |
| Watt et al 2013 ^(29)^ | England | Cornwall and Islington | Rural and urban | Children's centres | Local context- Differing organisational structures between the children's centres |
| Woodward et al 2015 ^(37, 38)^ | England | Leeds | Urban | Primary schools | NA |
